# Visual Sensitivities in Toddlers’ Hands Provide a Foundation for Shape Perception

**DOI:** 10.1111/desc.70291

**Published:** 2026-10-07

**Authors:** Alfredo F. Pereira, Thomas Endejan, Linda B. Smith

**Affiliations:** ^1^ Center for Psychology University of Porto Porto Portugal; ^2^ Psychological and Brain Sciences Indiana University Bloomington Indiana USA

**Keywords:** 3D shape, active vision, development, object‐centered representations, object recognition, vision

## Abstract

Human competencies in object recognition and category generalization implicate representations of the 3D geometry of objects. There is no accepted account of how such object‐centric representations are formed. Toddlers’ manual and visual exploration of objects is strongly associated with their emerging abilities in object recognition and category generalization, although there is limited understanding of the processes that underlie this association. Building on Marr and Nishihara's seminal theory of shape, we proposed (1) that toddlers actively select views that optimize fundamental visual sensitivities for long extents and for edges oriented horizontally and vertically and (2) that these biased views result in the axis of the object's maximum elongation, the major axis, being stably present in the variable 2D shapes projected from the object to the eye. We analyzed the visual properties of toddlers’ (*n* = 90, 12–38 months) egocentric views as they manipulated objects. We observed a highly biased sampling of object views in which the major axis of the physical object is near maximal extension and oriented near horizontal or vertical, two properties that make the major axis stably determinable in the variable 2D shapes projected to the eye. The findings show how fundamental low‐level visual sensitivities may play a critical developmental role in biasing behaviors that bias the input in ways that make hard computational problems tractable. The findings also provide an empirical agenda for research focused on the development of object‐centric representations of 3D shape.

**Summary:**

- We examined the visual properties of toddlers’ egocentric views of objects.
- We found that the generated views were a selective sampling of possible views that optimized foundational low‐level visual properties for shape perception.
- The findings implicate strong intrinsic constraints on toddlers’ visual experiences of object views.
- The biases in the toddler generated views are consistent with class theories of object‐centric representations of 3‐dimensional geometry.

## 1 Introduction

Humans recognize common objects from unusual views. We also recognize instances of categories as having the same 3‐dimensional (3D) geometry despite considerable differences in their individual shapes (e.g., Ayzenberg and Behrmann 2022; Biederman 1987). These competencies, illustrated in Figure 1A, implicate abstract representations of the 3D geometry of objects. Advancing behavioral, neural, and computational findings support this conclusion (e.g., Azynberg and Berhmann 2022; Bonnen et al. 2021). Traditional theories (Biederman 1987; Marr 1982) explained these phenomena in terms of object‐centered representations organized by a coordinate system intrinsic to the physical object (Figure 1B). There is no consensus account of how perceivers could form such representations from the projected 2D images received at the eye (e.g., Ayzenberg and Behrmann 2022; German and Jacobs 2020; Goodale and Milner 2023; Sun et al. 2021). The theoretical barrier is that the physical shape an object is perceived as having a stable essential shape but the images projected to the eye are highly variable (Sun et al. 2021). The task of finding the intrinsic coordinate system from the 2D projections of a 3D object (Figure 1A) has proven a hard problem to solve, despite considerable effort (e.g., German and Jacobs 2020; Marr and Nishihara 1978; Sun et al. 2021). Recently, Sun et al. (2021) raised the concern that without some pre‐specification of the intrinsic axes, object‐centric representations might not be learnable from the varied 2D projections. Marr and Nishihara (1978) originally proposed that the solution might lie in constructing 3D representations from a biased subsample of views. Here we show that toddlers’ active visual explorations of an object generate a subsample of all possible views and that this biased subsample stabilizes a critical property of the object's 3D geometry.

**FIGURE 1:**
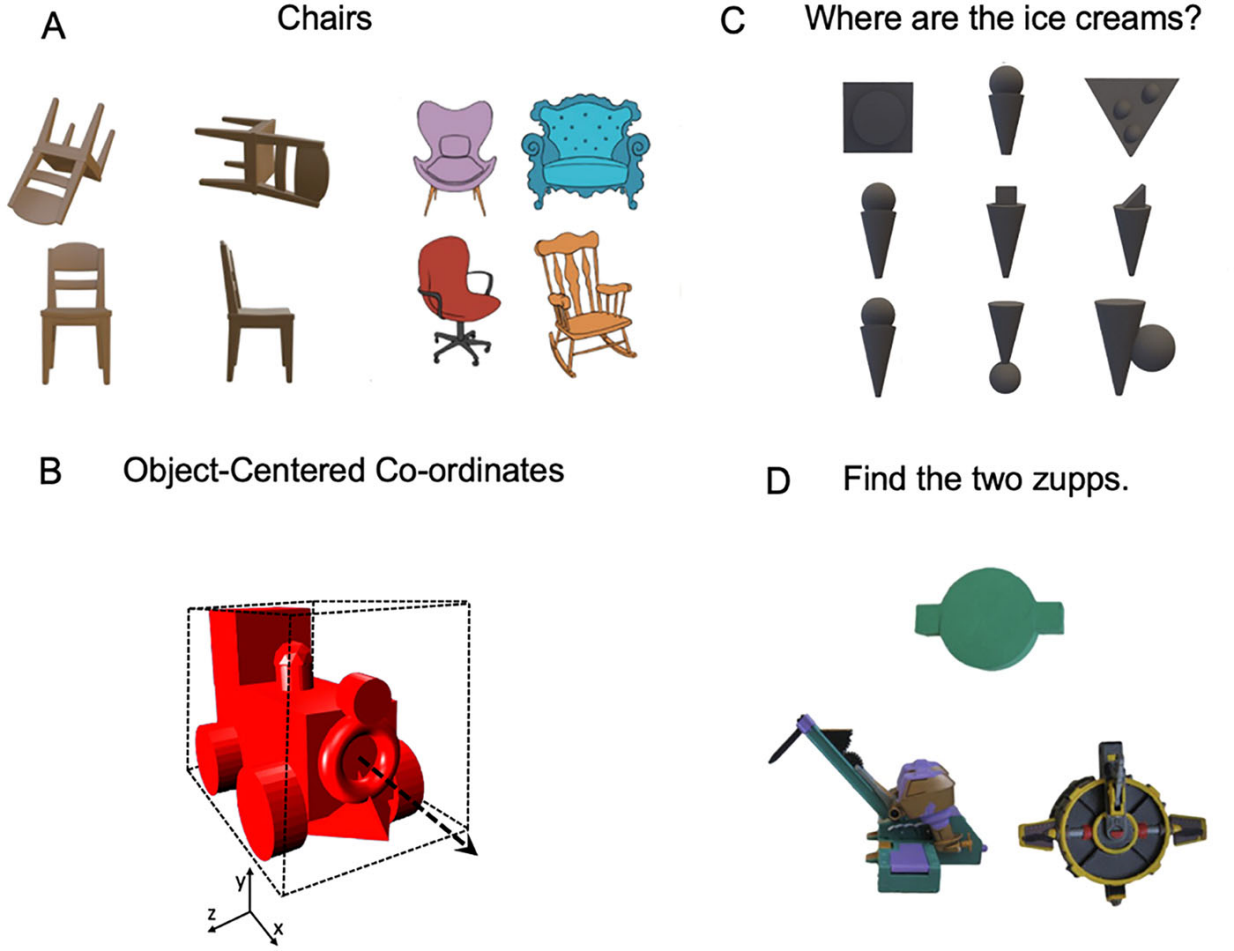
Competencies in human shape perception. (A) Individual objects are readily recognized from unusual views and different instances of the same category are perceived as shape similar despite differences in shape details. (B) Illustration of the object‐intrinsic coordinate system. The coordinate axes defined in terms of a tight‐fitting cuboid, the axis of maximal elongation of the cuboid, the major axis, is indicated by the heavy dashed line. The origin of the three axes lies at the intersection of the three axes. (C) Toddlers recognize common objects from sparse representations of 3D shape in terms of the relational organization of a few major parts (figure created from stimuli used in Augustine et al. 2011). (D) Toddlers generalize object names to new instances from a sparse representation of 3D shape (figure created from stimuli used by Son et al. 2008).

We focus on toddlers’ egocentric visual experiences because the extant evidence indicates that the period between children's first and third birthdays is one of rapid advancement in competencies that implicate representations of 3D object shape. These advances include, as illustrated in Figure 1C,D, recognition of objects from minimalist 3D caricatures of part structure consistent with Biederman's (1987) object‐centric theory of shape perception (Smith 2003; Augustine et al. 2011), the emergence of one‐shot adult‐like category generalization by shape (e.g., Son et al. 2008; Yee et al. 2012), and the generalization of object names to pictures of unfamiliar instances (e.g., Garrison et al. 2020).

The developmental period between the first and third birthday is also one in which toddlers are intensely interested in manually acting on objects. Estimates indicate that toddlers spend one‐third to one‐half of awake time handling objects (e.g., Fausey et al. 2016; Herzberg et al. 2022; Swirbul et al. 2022). Active object manipulation by toddlers has been associated with their performances in tasks measuring shape representation, category generalization, and object name learning (e.g., Contino et al. 2025; Karasik et al. 2025; Kingo and Krøjgaard 2011; Smith 2005; Slone et al. 2019). These facts suggest that the visual properties of toddler‐generated views may contain key information relevant to the development of 3D shape representations.

A variety of experimental studies using different methods including forced choice judgments as well as self‐generated views show that toddlers prefer some object views over others (e.g., Ghent 1961; James, Jones, Smith, et al. 2014; James, Jones, Swain, et al. 2014; Pereira et al. 2010; see Ayzenberg and Berhmann 2024, for review) but the visual properties of those biased views have not been well‐linked to processes that could build an object‐centric representation from variable 2D images. In their foundational theory of object‐centric representations, Marr and Nishihara (1978) and Marr (1982) made two proposals that provide the rational for the present study: they proposed that some object views are more relevant than others for building internal representations of the 3D geometry of objects, and they specifically suggested that views in which the major axis was maximally elongated would be optimal and present non‐accidental visual properties critical to forming shape representations. Accordingly, we asked if the object views that toddlers show themselves are selective and if, within that selectivity, the major axis is identifiable in the variable 2D images. As shown in Figure 1B, the major axis—the most elongated axis of the physical object—provides a potential reference frame for combining views, is the basis for defining the three orthogonal object‐centric axes and is the proposed representational backbone for a hierarchical organization of parts (Biederman 1987) and their symmetries (Blum 1973, Marr 1982). Accordingly, the present study was designed to answer this question: Do toddlers generate a biased sampling of views that makes the major axis readily discoverable in the visual properties of the projected 2D images?

The views and 2D images generated by toddlers depend, moment to moment, on the spatial relation between the eyes and the object, and thus on what toddlers do with their bodies. The hypotheses tested in this paper concern the spatial relations between the sensors and the object and the visual properties of toddler‐generated views, but not the specific bodily actions that create the spatial relations between eye and physical object. In the results, we present preliminary descriptive evidence concerning one potentially relevant bodily action—the number of hands in contact with the object. These descriptive results provide initial insights for future research on possible dependencies between body, actions, and the visual properties of self‐generated views.

### 1.1 Hypotheses

The perception of object shape begins with an object in the world that projects a momentary and potentially quite variable 2D shape to the eye. To be clear in presenting our rationale and hypotheses, we use the word “view” to refer to the spatial relation of the object to the sensors in terms of the major axis, a stable 3D property of a physical object. We will use the words “image” and “2D shape” to refer to images projected to the eye. We use the term *major axis* to refer to the most elongated axis of the physical object as illustrated in Figure 2A, and the term *main axis* to refer to the longest extent of the projected shape, as illustrated in Figure 2B and defined below.

**FIGURE 2:**
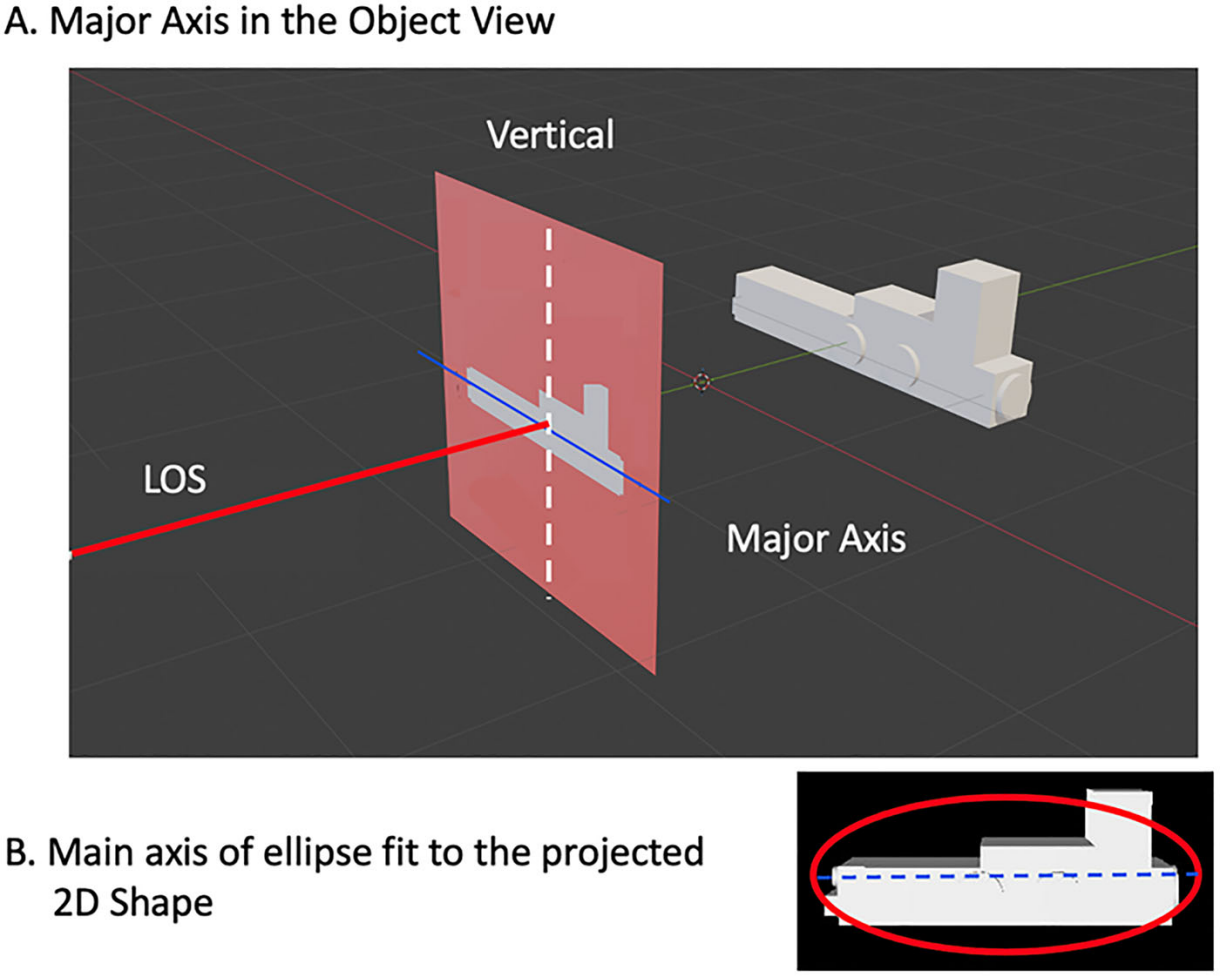
Object view and the projected 2D shape. (A) We measured the object‐centric view in terms of the angle of the major axis—blue line—to the line of sight (LOS, red line). When the major axis is perpendicular to the LOS, the projected extent of the axis in the received image in the picture plane (pink) is maximal. We also measured the orientation of the major axis within the picture plane relative to vertical (dashed white line). (B) We determined the main axis of the project 2D shape by fitting a 2nd moment ellipse to the shape and determined the orientation of the main axis (dashed blue line) of the ellipse in the picture plane. If the major axis is nearly fully extended its projection will correspond to the main axis of the fit ellipse as shown in (B). We also measured the orientations of local edges (not shown) in the projected image.

If the major axis of the physical object, a theoretically foundational property for object‐centric representations, is to be discovered by the visual system, it must be discoverable in the projected 2D shapes. We provide evidence for three interconnected hypotheses relevant to making this conclusion.

#### 1.1.1 Hypothesis Views: Extent and Orientations of the Major Axis

The human visual system is characterized by a set of sensitivities (Geisler 2008) that act as saliences, attracting and holding visual attention and optimizing the extraction of visual information (for reviews, see Smith and Karmazyn‐Raz 2026; Wolfe and Horowitz 2004). Two well‐documented visual sensitivities, for longer over shorter extents (e.g., Boutsen and Marendaz 2008), and for extents oriented horizontally and vertical rather than at oblique (e.g., Appelle 1972), are likely candidates for properties that could stabilize the major axis in a subset of projected images from the object. As illustrated in Figure 1A, Hypothesis Views proposes (1) that toddlers bias spatial orientations of the object relative to the eyes so that the major axis is near perpendicular to the line of sight (LOS). This spatial relation maximally extends the length of the longest physical axis of the object. Hypothesis Views also proposes (2) that toddlers orient the major axis so that it near horizontal or near vertical with respect to the toddler's head‐centered view. Note, in infants and young children, the advantages of horizontal and vertical in the extraction of visual information is relative to head‐centered vertical not gravity as it is in adults (See Jouen 1985; Petroff et al. 2025).

#### 1.1.2 Hypothesis 2D Shape

We measured three properties of the 2D object shapes in the head camera images without regard to the major axis and the geometrical properties of the physical object. We first determined the main axis, or most elongated axis of the 2D shape. The main axis is well documented to influence 2D shape perception (Sekuler 1996). Second, we measured the orientation of the main axis relative to head‐centered vertical as orientations of the longest extent of a 2D shape strongly influence the perceived shape, comparison, and categorization (Rock 1974; Houbben and Vannuscorps 2026). Third, we measured the local edge orientations of the projected shape as edges and their orientations, the first stage of information extraction for shape perception. As illustrated in Figure 2B, we fit a second‐moment ellipse to the 2D shape and measured the orientation of the main axis of the ellipse relative to vertical. At the local level, we measured the distribution of local edge orientations. By Hypothesis 2D Shape, toddlers are predicted to generate a biased sample of images in which the elongated axis of the 2D shape (the main axis of the fit ellipse) and the local edges in the image will be oriented horizontally or vertically with respect to the head‐centered view.

#### 1.1.3 Hypothesis Major‐Minor Axis

The key hypothesis is that the major axis of the physical object is stably detectable in the biased toddler‐generated views because the major axis of the physical object is often the main axis of the projected shape. If toddler‐generated views are biased such that the extent of the axis of greatest elongation of the physical object is near maximal extent, then the major axis will correspond to the main axis of the projected 2D shape. As a consequence, the measured orientations of the major axis of the physical object and the main axis of the projected shape relative to vertical in the head‐centered view should correspond.

## 2 Method

### 2.1 Corpus of Images

Our analytic approach is modeled after the literature on the scene statistics of photographs of the physical world, an approach which has led to significant advances in the human vision (Geisler 2008). However, our images are captured by head‐cameras worn by toddlers handling objects not by photographers pointing cameras at the world. We selected the images for this study from a larger corpus of egocentric images captured by toddlers visually exploring objects. For the present analyses, we included contexts in which one object was explored at a time. We selected four objects for analyses based on their shape properties as described below. A small set of images in the larger corpus were included in a prior published study (Pereira, James, Jones & Smith, 2010), and a portion of that data are included in the present analyses (OSF Pereira, Endejan & Smith, 2026). All data in the dataset were collected in accordance with Indiana University Protocol 902000021. The sample of children in the dataset represents the demographics of the county and state in which the data were collected: The toddlers were recruited from an opt‐in database for developmental research, which was advertised at outreach events for infants, children, and their parents. Children contributing to the corpus of images represent the demographics of Monroe County, Indiana which includes a large university community (68% European American, 9% African American, 7% Asian American, 8% Latino, 7% Mixed race).

### 2.2 Objects

The four objects selected for this study are shown in Figure 3. They were selected to provide a strong test of the hypotheses by being relatively simple in shape and presenting a major axis that was both the axis of elongation and an axis of symmetry (as these two properties of common physical objects often correspond, Marr and Nishihara 1978) and local geometries that offered many perpendicular edges. If longer extents (e.g., Boutsen and Marendaz 2001), and horizontal and vertical orientations of edges and extents, are biased in toddler‐generated views, they should be readily evident for these object geometries. The four objects also had object‐centered axes that differed in their relative extent, as detailed in Figure 3, since it seemed possible that a significant length advantage of the major axis over the other two axes might be a factor in linking the major axis of the physical object to the main axis of the projected images. The object‐centric axes and their extents were measured by creating a bounding box tangent to each side of the object, yielding a cuboid from which the three axes and the object's extents on those axes were determined (as in Figure 1B).

**FIGURE 3:**
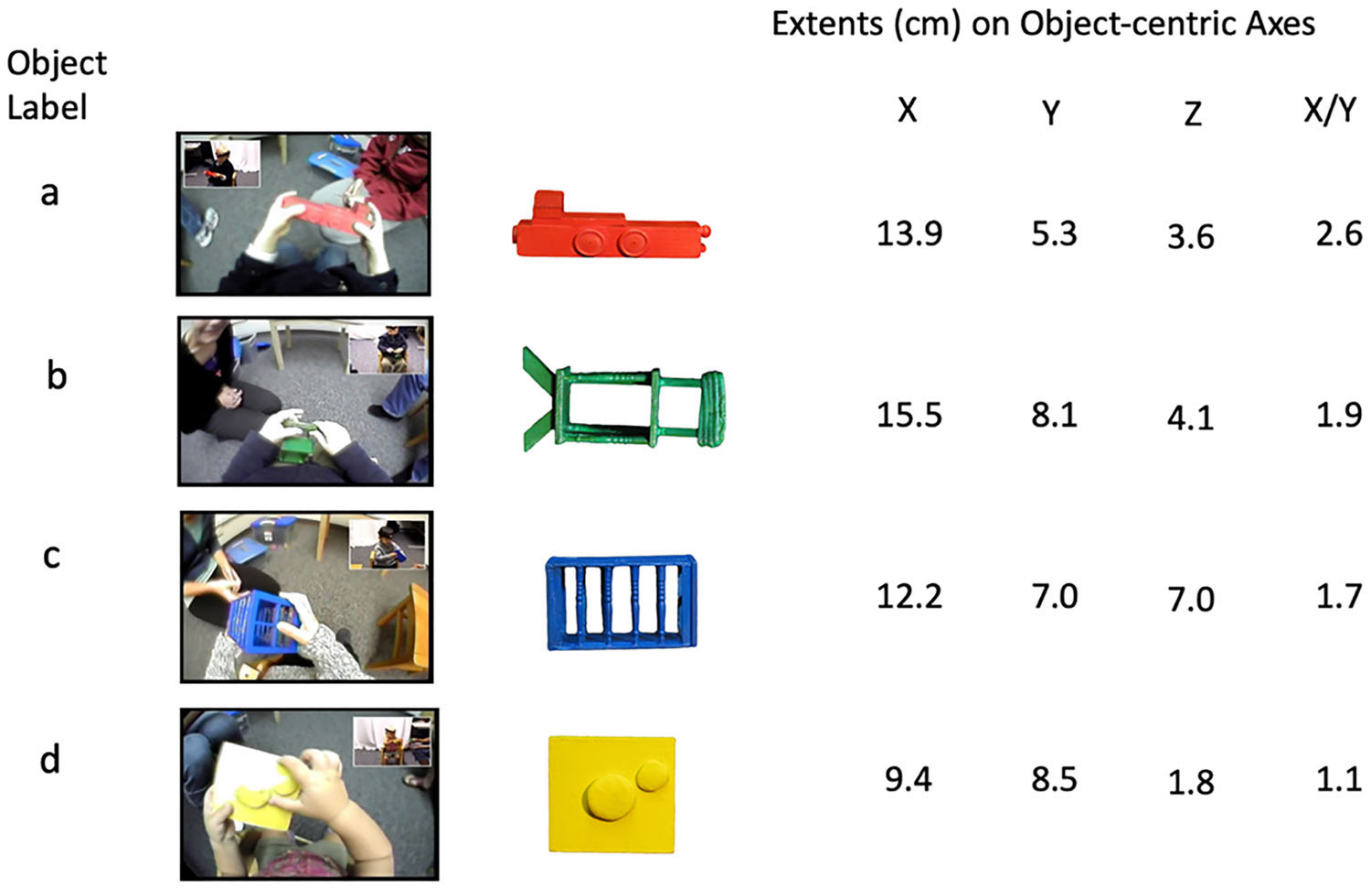
Objects and their extents on the object‐centric axes. Shown for each of the four objects, labeled a, b, c, and d, is a head camera image, a photograph of the object, and the extents (cm) of the three object‐centric axes (X, Y, Z) and the ratio of the extent of greatest elongation to the next greatest (X/Y).

### 2.3 Participants Contributing Images for this Paper

Ninety children, mean age M = 23.9 months, range 12–38 months (42 female) contributed data to the present analyses. Not all children contributed data for all four of the selected objects (a listing of participants by age and objects explored is available at OSF Pereira et al., 2026).

### 2.4 Head‐Camera

The camera was a WATEC model WAT‐230A with 492 effective image frame pixels, weighing 30 g and measuring 36 mm × 30 mm × 30 mm. The lens used was a WATEC model 1920BC‐5, with a focal length of f1.9 and an angle of view of 115.2° on the horizontal and 83.7° on the vertical. The camera recorded at 30 Hz. The camera was attached to a headband that held the camera low on the forehead directly above the nose. The camera captures the images in front of toddler faces but not where the toddler is looking within that image. Infants and toddlers tend to orient eyes and heads in the same direction bringing them into alignment within 500 ms when shifting attention (Yoshida and Smith 2008; Mendez et al. 2024; Borjon et al. 2021). When holding objects, they tend to center the held object in the field of view and at midline of the body (Bambach et al. 2016; Borjon et al. 2021). Studies using head‐mounted eye‐tracking demonstrate that in these contexts 80% of gaze by infants and children between 1 and 3 years of age falls within 20° of the horizontal and vertical center of the head‐camera image (e.g., Bambach et al. 2016; Borjon et al. 2021).

### 2.5 Procedure for Data Collection

The child sat on a small chair and the parent sat nearby. A removable table with a push‐button pop‐up toy was placed close to the child to assist in placement of the head camera on the child's head. One experimenter directed the child's attention to the pop‐up toy while a second experimenter placed the head camera on the child's head. This was done in one movement. The parent assisted by placing his/her hands above the child's hands, gently preventing any movement of the hands to the child's head as the head camera was put on. With the child engaged in pushing buttons on the pop‐up toy, the head camera was adjusted so that when the child was pushing a button, that button was in the center of the head‐camera field. The table was then removed so that when the child was given an object to explore there was no support for the toy other than the child's own hands. The child was given one object at time. The experimenter made encouraging and neutral statements (e.g., “Look!”; “Look at that!”). The trial ended when the child lost interest in the object.

### 2.6 Analyzed Data

The data used in the study consists of 232 individual bouts of object manipulation (an uninterrupted sequence of frames from the same object) and these lasted on average M = 15.79 s, SD = 14.65 s. The duration is consistent with other studies in which children manually and visually explored objects (James, Jones, Smith, et al. 2014; Pereira et al. 2010; Slone et al. 2019) and is higher than the operational definition of sustained attention (3 s) commonly used in studies of object manipulation and visual exploration in toddlers (e.g., Mendez et al. 2024). Active visual and manual exploration of an object that lasts longer than 3 s predicts learning about that object (Pereira et al. 2014). Images from the head‐camera videos were sampled at 1 Hz and this generated *N* = 3664 images for analysis. The number of images analyzed for the four objects was (objects a–d, Figure 3) 786, 639, 1548, and 691.

### 2.7 Coding of Object View

A custom‐built application was developed for human coders to assist in object pose estimation. This application has two panels: in the left panel, the coder could see a video frame of a toddler's held object; in the right panel, the coder could rotate a 3D rendering of the object to match the orientation in the head camera image. The orientation of the 3D object at that point was recorded by the application. The coding application used a standard coordinate system: the X axis on the horizontal of the screen, positive toward the right; the Y axis on the vertical of the screen, positive toward up; and the Z axis positive toward the viewer. The XY plane in this coordinate system corresponds to the picture plane. The line of sight (LOS) was represented by the unit vector (0, 0, –1). Object rotation was represented using Euler angles, and the sequence used was YZX. Before starting any rotation, the to‐be‐rotated 3D rendition was presented in the same initial orientation, equivalent to the Euler angle rotation sequence of (0°, 0°, 0°). Applying a particular sequence of Euler angles to this vector, rotated the major axis of the elongation vector into the position in 3D space that best matched the video frame. One coder was the primary coder for each of the four objects. Two coders independently coded 20% of the images and matched within ± 3° on each of the three axes (considered independently) 90% of the time; the coding of the primary coder was used in all cases of disagreement.

### 2.8 3D Renderings

Renderings were created that matched the pose of the 3664 head‐camera images. These renderings were used for all analyses. The renderings were made using a custom Python script and the PyOpenGL package. Image size of renderings was 800 × 600 pixels and no scaling was done to the object. The camera used default settings and was placed in the Z axis so that the final rendering was deemed correct. Objects were rendered with a flat gray color and a small amount of specular reflection in a lighter gray. The background was black. Each rendering was saved into an image file.

### 2.9 Measures of Views

The unsigned angle between the vector representing the LOS and the vector representing the major axis of elongation was calculated, and then converted to a 0°–90° range, from parallel to perpendicular to the LOS. To measure the orientation of the major axis within the picture plane, the unit vector that represented the major axis was projected onto the picture plane (in our coordinate system, the XY plane). We then computed the unsigned angle between the projected vector and the picture plane's vertical. This angle was converted to a 0°–90° range, with 0 indicating head‐centered vertical. We do not report measures of orientation with respect to adult canonical views because toddlers do not consistently hold and explore objects in ways that maintain adult canonical views (see Bambach et al. 2018).

### 2.10 Measures of 2D Shape

We measured two properties of the 2D shape without regard to the 3D structure of the object (Figure 2B). Using a custom MATLAB script, we computed the orientation of the second‐moment ellipse fit to the set of all object pixels. This provides a measure of the orientation of the longest extent of the projected 2D shape. The second‐moment ellipse was computed using the pure moments method (Gonzalez and Wood 2002). This angle ranged from 0°, when aligned vertically in the picture plane, to 90°, when aligned horizontally. We computed the distribution of edge orientations in the rendered image using the method and source code described in (Redies et al. 2017).

### 2.11 Null Hypothesis Data

A null set of renderings was created using a custom Python script. We generated random rotations in 3D (*N* = 8000) using the scipy.spatial.transform package. For the random number generator we selected the MT19937 generator in the numpy package.

### 2.12 Coding of Holding With One or Two Hands

Studies of toddler object manipulation indicate that they often hold, manipulate, and visually explore objects with preferences for symmetric positions of the two arms and with the two hands operating symmetrically or with distinct coordinated actions (e.g., Fagard and Marks 2000, Needham and Nelson 2023; Franchak and Adolph 2024). The manner of holding also varies with the shapes of objects (e.g., Ruff 1984; Thompson et al. 2025). Because two‐handed versus one‐handed holding could play a role in the self‐generated views, we coded a subsample of the images (*n* = 2986, 81%) for whether one or two hands was in contact with object. Two human coders coded each head camera image choosing one of the following three alternatives: two hands were in contact with the object, one hand was in contact, or that decision was not determinable from the head camera image. The two coders agreed on 86.0% of the images. Disagreements were decided by a third independent coder.

### 2.13 Data Analysis

Our analytic approach, consistent with a scene statistics approach, is conducted at the level of the corpus of all images without regard to participant or object. Subsequent to the main analyses, we provide descriptive statistics with respect to the age of the child generating the images, the effect of the specific object on those statistics, and whether the object was held with one hand or two.

### 2.14 Data and Code Availability

The data for the study is available on OSF (Pereira et al. 2026). This includes the toddler dataset used in this study, the dataset generated by simulation for the null hypothesis, and the image corpus of renderings generated from the 3D object pose data. All code is available on OSF (Pereira et al. 2026).

## 3 Results

### 3.1 Hypothesis Views

In the context of the present task, children's behaviors—head, posture, and hand actions—determine the momentary spatial relation of the object to the head. Hypothesis Views proposes that toddlers behaviors bias this spatial relation so that the major axis is near perpendicular to the toddler's LOS and that they orient the object with respect to the head so the major axis, the most elongated extent of the physical object, is oriented horizontally or vertically in the head‐centered view. Figure 4 A,B shows strong support for both predicted outcomes. The distributions of orientations of the major axis relative to perpendicularity to the LOS differed from that the simulated null distribution (Figure 4A), K–S statistic D = 0.231, *p <* 0.001, with the observed data showing more near perpendicular orientations (and thus near maximal extension) than the simulated null. The simulated null distribution is not uniform but reflects a mathematical fact of projections from a 3D object to a plane: a uniform sampling of the orientations of vectors from the unit sphere does not yield a uniform distribution of orientations of those vectors to the plane but yields more orientations near perpendicular than parallel (Hartley and Zisserman 2003). Thus, the toddler data is an exaggerated version of the null. The proportion of toddler views in which the major axis is within 10° of perpendicular to the LOS and thus near maximal extent is 37%; for the null it is 17%. Toddlers orient the object with respect to their head in ways that bias long (near maximal) extents of the major axis in the projected image, a bias consistent with human greater sensitivity to longer over shorter extents and thus relevant to visual information extraction and forming shape representations.

**FIGURE 4:**
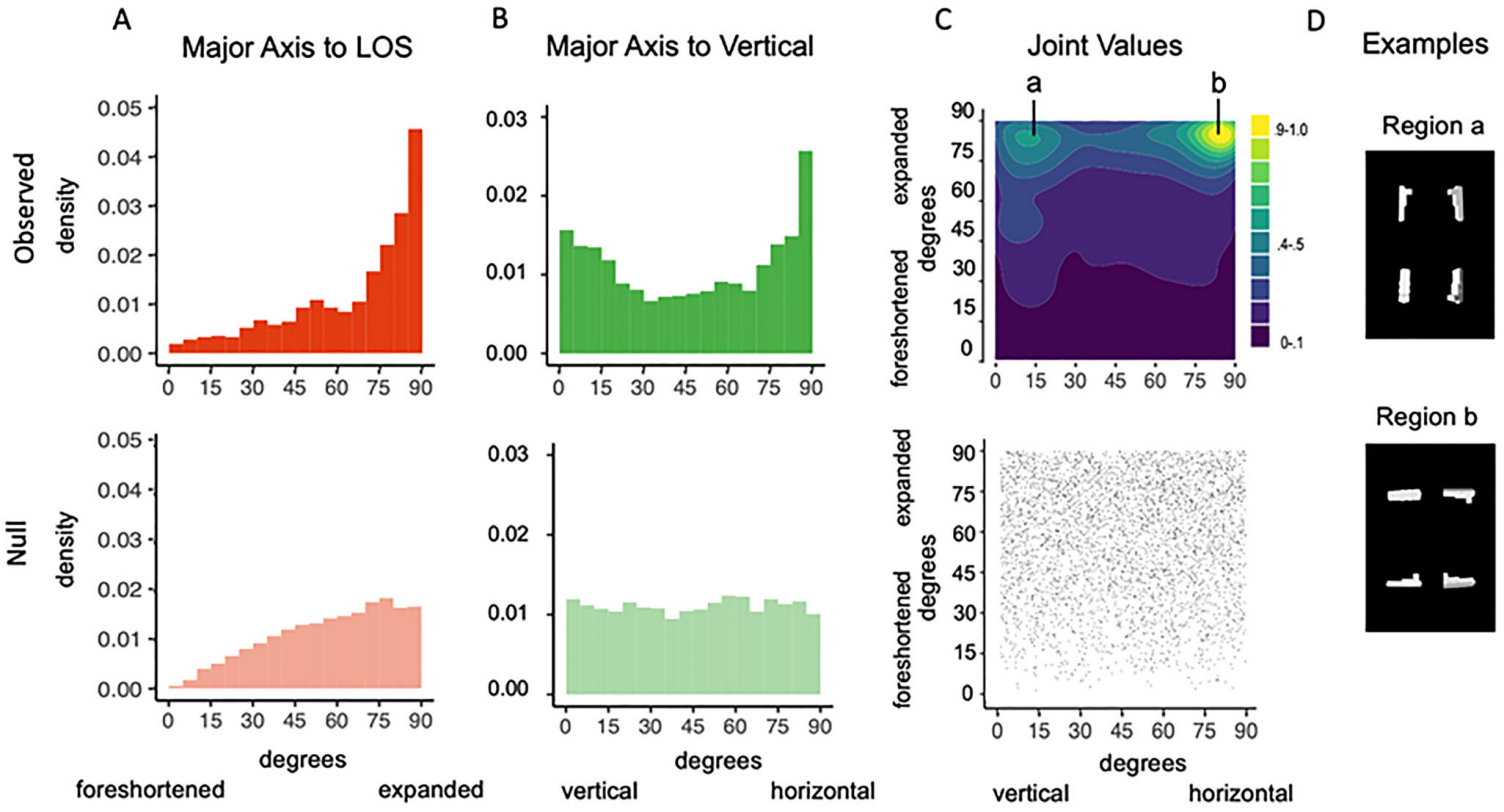
The views toddlers show themselves. (A) Density histogram of the angle of the major axis to the line of sight (LOS) for the Observed (red) and Null (pale red) data, showing the bias for views in the major axis is near maximal extent. (B) Density histogram of the angle of the major axis to vertical in the picture plane, for the Observed (green) and the Null (pale green) data, showing a bias for horizontal and vertical orientations of the main axis. (C) Top: Heat map of frequency of joint values of angles of major axis to LOS and major axis to vertical in the picture plane showing the selectivity of toddler sampled views. Bottom: scatterplot of the same joint values for the randomly generated Null data. (D) Example views of objects in the two densest regions of (5) joint values of orientation of the Major Axis to LOS and to vertical for the Observed data. The dominant views are rotations around the major axis oriented vertically (Region a) or horizontally (Region b).

**FIGURE 5:**
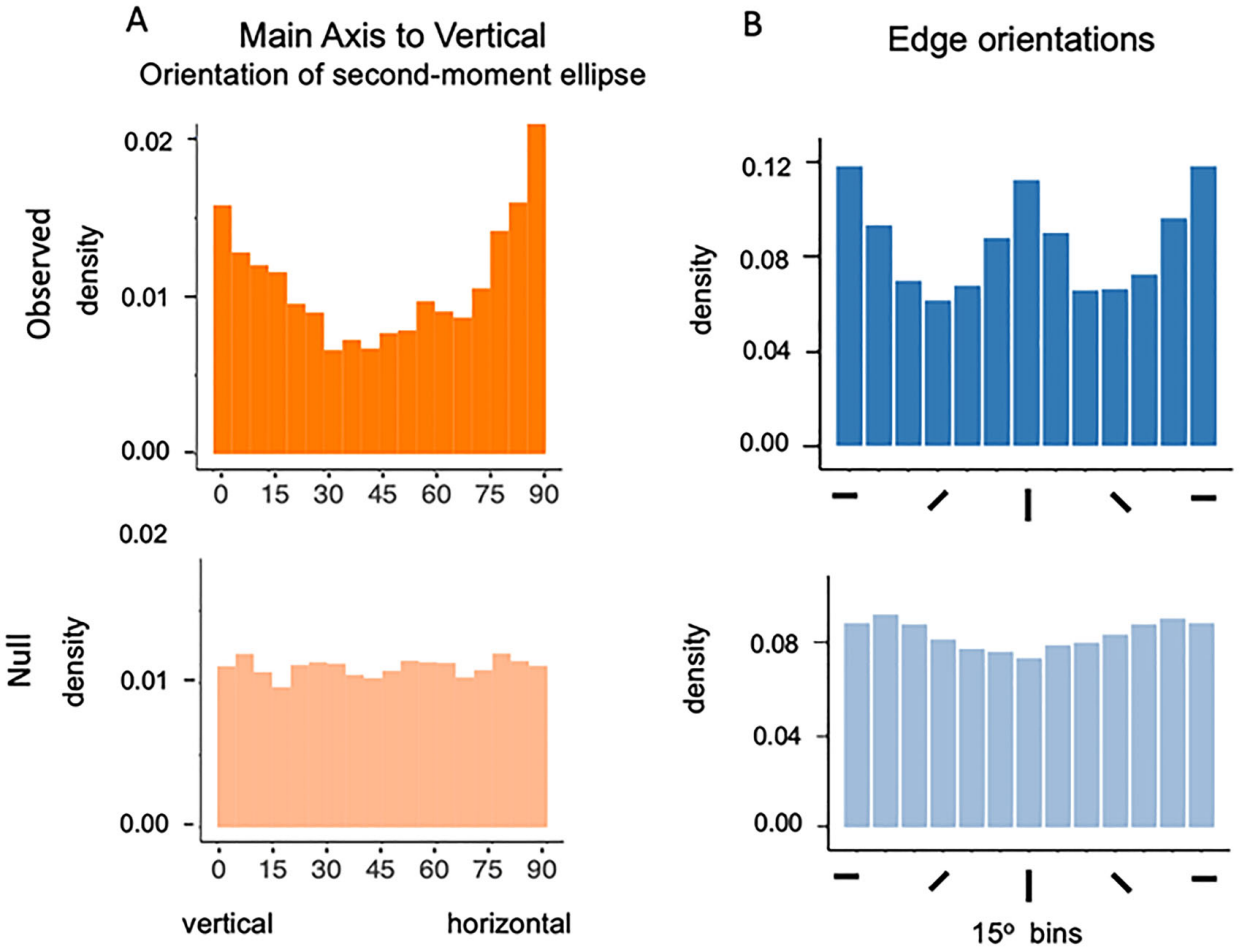
The 2D shapes toddlers show themselves. (A) Density histogram of orientation of the main axis of the second‐moment ellipse from vertical (0°) to horizontal (90°) in each toddler‐generated image, Observed data, and in the randomly generated Null. The orientation of the ellipse is strongly biased to horizontal and vertical orientations. (B) Density histogram of local edge orientations of the object in each toddler‐generated image, Observed data, and the randomly generated Null. The orientation of local edges is also biased to horizontal and vertical.

Toddlers also biased horizontal and vertical orientations of the major axis in the head centered view (Figure 4B). The frequency distribution of orientations of the major axis differs from the simulated random selection of views that is the null, K–S statistic D = 0.102, *p <* 0.001. In the toddler data, horizontal orientations are more frequent than vertical ones, with 20% of frames within 10° of horizontal within the picture plane, and 15% of frames within 10° of vertical, exact binomial test for probability of success 0.5, *p <* 0.001. We return to this asymmetry when we discuss object effects. The bias to orient the object so that the major axis is oriented in line with the cardinal orientations is consistent with human greater visual sensitivity to edges oriented horizontally or vertically rather than oblique orientations and thus relevant to visual information extraction and forming shape representations.

As shown in Figure 4C, toddlers freely generate a highly constrained sub‐sample of possible views as shown in Figure 4C. As illustrated in Figure 4D, the sampled views are principally rotations around a nearly fully extended major axis that is oriented near horizontal or near vertical in the head‐centered view.

### 3.2 Hypothesis 2D Shape

The 2D images received at the eye, not the physical object, are the sensory input for vision. Consistent with human privileged sensitivities to the cardinal orientations and as shown in Figure 5, both the main axis (most elongated) of the ellipse fit to the projected shape and the local edges of the projected shape were biased to horizontal and vertical orientations. The distribution of the toddler‐generated orientations of the second‐moment ellipse differed from that of the null data set, K–S statistic D = 0.105, *p <* 0.001. In addition, the main axis, like the projection of the major axis of the object, was more strongly biased to horizontal than to vertical, with 20% of frames within 10° of horizontal and 14% of frames within 10° of vertical, exact binomial test for probability of success 0.5, *p <* 0.001. Toddlers’ views were also biased with respect to the distribution of orientations of local edges with 12% of all edges in the frames being horizontal and 11% being vertical. The total proportion of horizontal and vertical local edges in the toddler generated images, 23%, exceed the proportion in the null, 16%, 2‐sample test for equality of proportions, χ^2^(1, *N* = 11164) = 77.597, *p <* 0.001. These visual properties are relevant because humans, including infants (Jouen 1985), are better able to detect and discriminate edges and extents that are not oblique and because the main axes of a 2D shape, oriented in line with cardinal orientations, influence shape representations (e.g., Rock 1974; Houbben and Vannuscorps 2026). The results show that when actively engaged with an object, toddlers generate input is biased in favor these properties that benefit information extraction and shape perception.

### 3.3 Hypothesis Major‐Main Axes

As shown in Figure 6, when the major axis, a stable property of the physical object, is near perpendicular to the LOS, its extent is near its maximum and as a consequence, the major axis of the physical object is aligned with the orientation of the main axis of the projected 2D shape. We quantified, frame by frame, the correspondence of the orientation the major axis of the object and main axis of the fit ellipse (vertical to horizontal) in the head centered field of view for the observed toddler and simulated null data. As shown in Figure 6, in both the observed and null data, the main axis of the 2D shape is highly likely to correspond to the major axis.

**FIGURE 6:**
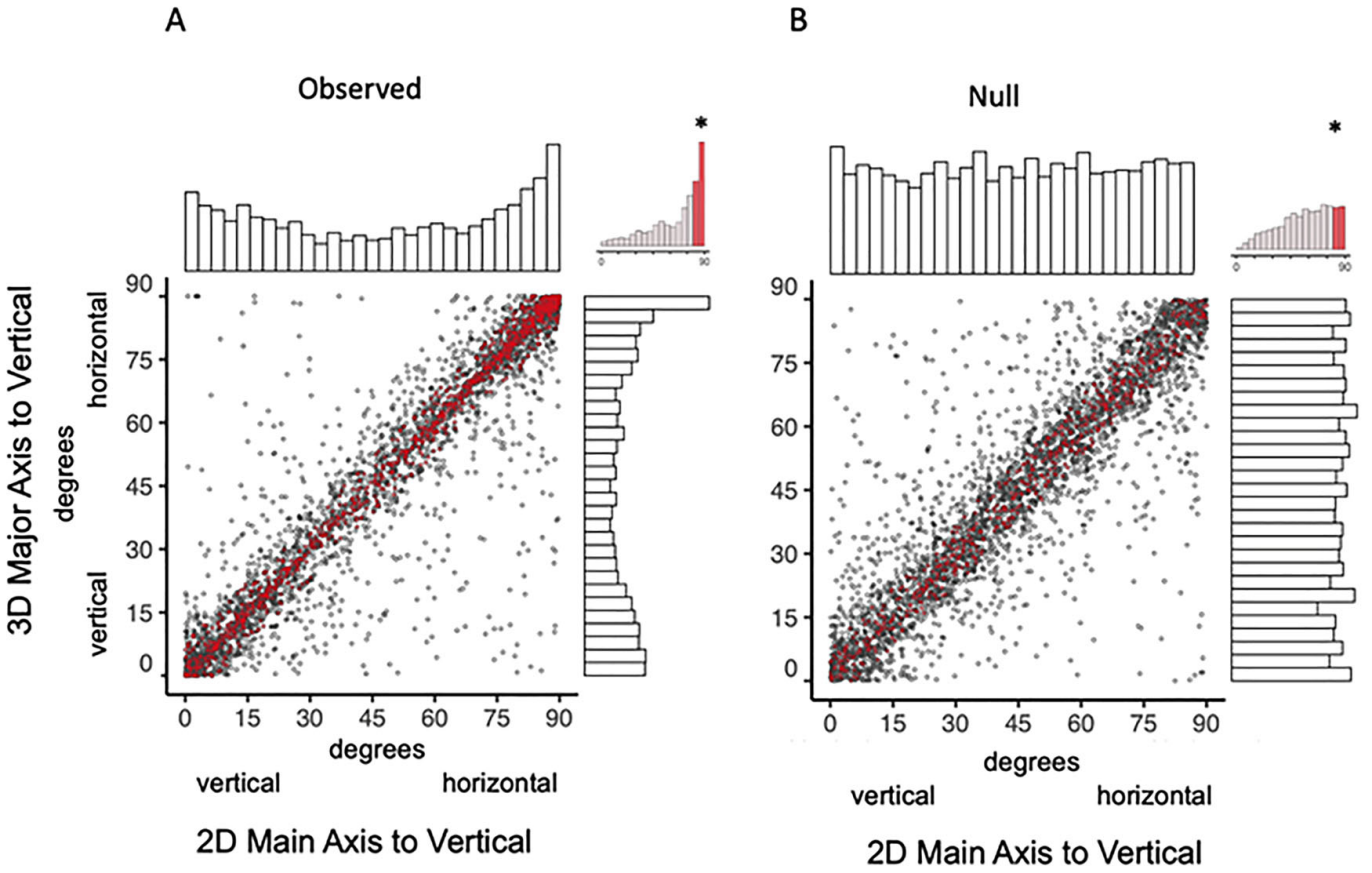
The correspondence of the major axis of the view to the main axis of the fit ellipse. Each dot indicates for a single image the joint values of the orientation (from horizontal to vertical) of the major axis and the main axis. The red dots indicate cases in which the major axis was ≤10° from perpendicular to the LOS and thus near maximum extent. The Null data show a random sampling of the 8000 rotations used to generate the null that equates the number of data points (3664 images) in the two figures. The distributions of orientations of the major axis of the object and main axis of the fit ellipse relative to vertical in the picture are shown on the corresponding X and Y axes. The inset frequency distribution, upper right of each panel with asterisk, shows the orientations of the major axis relative to the LOS. Both figures show a stronger correspondence between the orientations of the Major axis and the fit ellipse, but it is stronger in the toddler data and clustered near horizontal and vertical orientations.

The toddler data yields an extraordinarily high correlation between the orientations of the main and major axis relative to vertical, Spearman's correlation *r_s_* (3662) = 0.924, *p <* 0.001. For toddlers, the aligned orientations of the main and major axes within the head centered image fall along the line of maximal extent and are clustered in the regions of horizontal and vertical orientations. The null data also show a significant and strong correlation between the measured orientations relative to vertical of the main axis of the 2D shape and major axis of the 3D object; the Spearman's correlation *r_s_* (7998) = 0.896, *p <* 0.001) is only slightly weaker than in the toddler data. Without any bias on the part of infants, the physical world through the geometry of 3D projections to 2D planes, provides a direct statistical path to finding the projection of the major axis in the 2D images. This fact of geometry may be relevant to both evolutionary and developmental constraints on human visual object recognition, on object‐centric representations, and potentially on how toddlers spatially orient objects for viewing. Further quantification of the predictive relations between the major and main axes in both the observed and null data sets are available in the OSF (Pereira et al. 2026).

Critically, the toddler‐generated data have two further enhanced properties relative to the null that may benefit discovering the utility of the major axis for combining views and for representing object shape. First, the major axis of the object (and thus the main axis of the projected shape) is more often near maximal extent than the generated null images and thus, for the human perceiver, presents a more salient aspect of the 2D image (e.g., Boutsen and Marendaz 2001). Second, the major axis of the physical object and the corresponding main axis are clustered at two orientations in the picture plane, near horizontal and near vertical.

#### 3.3.1 Age Effects

The proportion of images in which the major axis was at or within 10° of perpendicular to the LOS and thus near maximal extent increased with continuous age (logistics mixed‐effects model, *p <* 0.002). The correlation between the frequency of near maximal extent and age, however, is quite small and accounts for very little of the variance (*R* ^2^ = 0.081). There were no age effects with respect to horizontal or vertical biases at the local edge level, for the major axis, nor for the main axis of the projected 2D shapes (*p* > 0.50 all comparisons). Although the age range of children participating is broad, from 12 to 38 months, the biases in toddler generated views appear stable during this period of development. The lack of developmental differences is not surprising if visual sensitivities to long extents and cardinal orientations are a motivating factor in children's spatial orientation of the objects relative to their head and eyes. These are core human visual sensitivities that attract gaze as evident in infant preferential looking behavior from the neo‐natal period forward (Kiorpes 2016, Anderson et al. 2024).

#### 3.3.2 Object Effects

Figure 7 shows the percentage of frames in which the major axis is near maximum extent (≤10° of perpendicular to the LOS) and the percentage of frames in which the major axis of the 3D object view and the orientation of the main axis of the 2D shape were near (≤10°) horizontal and vertical for each of the four objects. The overall pattern is highly similar for all objects; however, the small differences between objects are reliable. This fact implicates not‐yet‐identified factors associated with the individual objects. For the relation of the major axis to the LOS, objects a and b, the two objects with the greatest relative extent of the major axis over the remaining object‐centric axes, were less frequently fully extended than objects c and d (χ^2^(3) = 68.52, *p <* 0.001), opposite of our original conjecture that the greater the length advantage of the major over the other object centric axes, the stronger the bias in the views and projected shapes. Vertical orientations of the major axis (χ^2^(3) = 48.92, *p <* 0.001) also differed by object with vertical orientations more dominant for object b and horizontal orientations of the major axis more dominant for object were more frequent for objects b and d; horizontal orientations of the major axis were more frequent for objects c. These same patterns were observed for the orientations of the main axis of the fit ellipse (χ^2^(3) = 41.27, *p <* 0.001). There were no object differences in the distributions of horizontal and vertical orientations of local edges. Although all objects show the same overall biases, the small but reliable object differences indicate that there are factors influencing toddler‐generated views that were not measured in the present study. These may include ease of holding and rotating objects, the role of surface features, or potentially other not measured visual properties.

**FIGURE 7:**
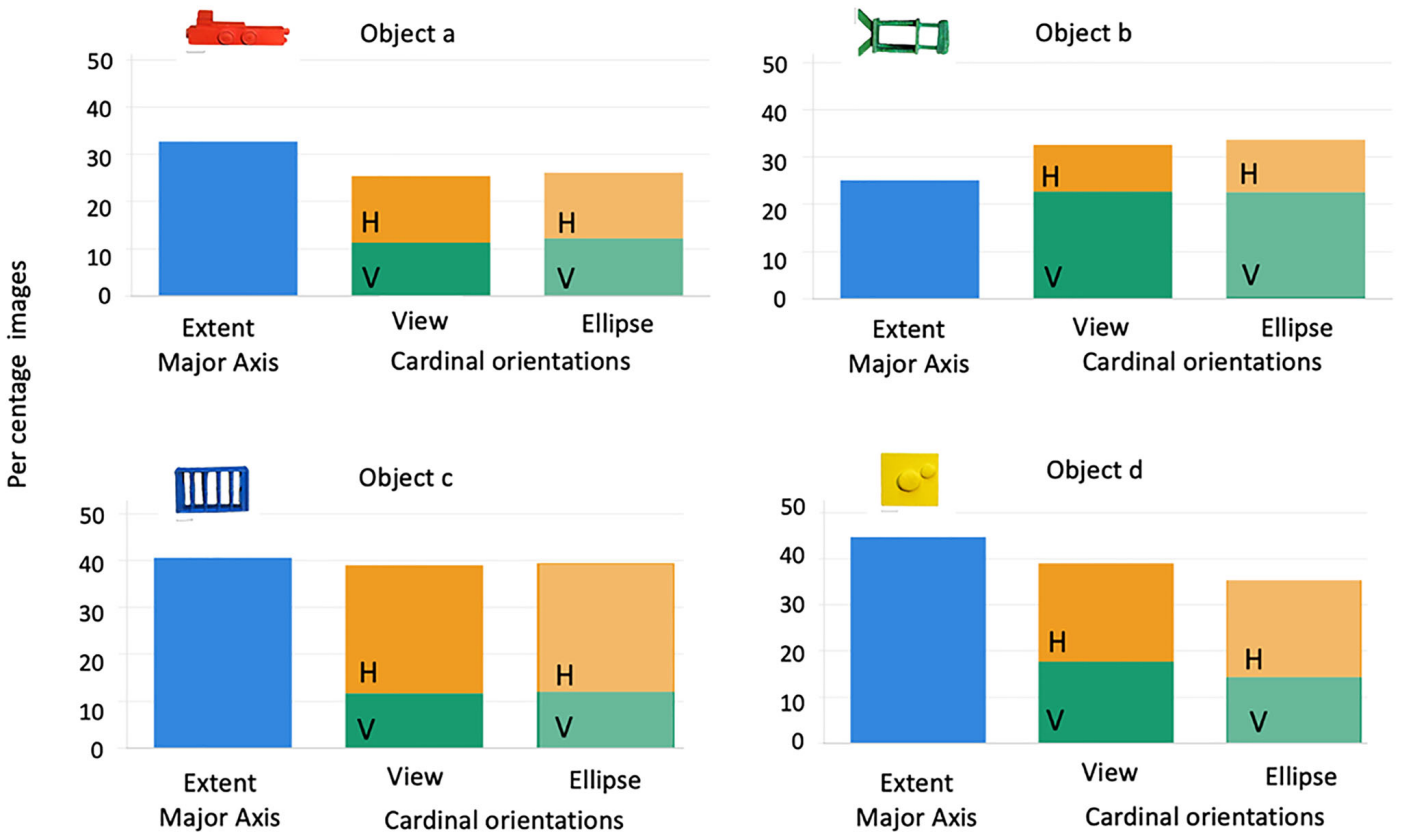
Object effects. Each panel shows the percentage of images in which the major axis was near perpendicular, ≤10°, and thus near maximal extent (blue), the percentage of images in which the major axis in the object view was oriented horizontally or vertically, (dark green and orange), and the percentage of images in which the fit ellipse was oriented horizontally or vertically (pale green and orange).

We also examined the frequency with which toddlers held the objects with one hand or both. Overall, two‐handed holds accounted for 65.1% of coded images, and one‐handed holds accounted for 33.2% of frames; in 1.7% of frames, the object was held too close to the toddler's face for the hands to be seen. Two‐handed holds dominated for all objects, but there were reliable differences, Pearson's χ^2^(3) = 21.342. Objects a and b, the two objects with the longest physical extent of the major axis relative to other object axes, were held with two hands less often, 60% and 59% respectively, whereas objects c and d were held with two hands 68% and 70% of the time, respectively. In brief, the two objects that were long and thin (with the global shape of a fat dowel) had a slightly but reliably smaller ratio of two‐handed holding vs. one‐handed holding.

When an object was held in both hands, it was more likely to be held such that the major axis was within 10° of perpendicular to the LOS and thus fully extended than when it was held at any other angle relative to the LOS, 70% versus 62%, Pearson's χ^2^ test with Yates' continuity correction, χ^2^(1) = 19.282, *p* < 0.001. Thus, although two‐handed holds are highly frequent in all the images, they are also associated with being held perpendicular to the LOS and maximally extended in the view. The likelihood of two‐handed holding also varies with orientation of the major axis, Pearson's χ^2^(2) = 88.902, *p* < 0.001. When the major axis was within 10° of horizontal, two hands held the object 79.3% of the time; when the major axis was within 10° of vertical, 68.7% of holding behavior involved both hands. For all remaining orientations, two‐handed holds accounted for 59% of the cases. Thus, two‐handed holds are also associated with stronger biases in the visual input for a major axis aligned with the cardinal orientations.

These descriptive findings are consistent with a number of testable hypotheses: Two‐handed holds dominate across the images and are clearly the overall preferred approach for toddlers during visual exploration of an object, and may be because two‐handed holds are easier to control. Alternatively, or in addition, longer, thinner objects (a and b), which are more likely to be grasped with one hand and present are more likely to be held in one hand and, as a consequence, present noisier orientation data. The idea that controlling the hands might limit preferred views is supported by prior findings (James, Jones, Swain, et al. 2014) showing stronger biases in views sampled when the object was placed in a transparent sphere such that the hand actions were equipotential for all views than when the object was held directly. James, Jones, Swain, et al. (2014) hypothesized that visual properties biased the generated views but that awkward and noisy manipulations were a limiting factor in toddler‐generated views.

In brief, the overall pattern in the observed data is for strong biases with respect to the spatial relation of the object to the toddlers’ head, for the resulting object views, for projected shapes with the main axis aligned to the major axis of the physical object, for cardinal orientations, and for holding objects with two hands. As a final analysis, we assessed the independence of the following factors using a log‐linear model: number of hands in contact with the object, major axis oriented within 10° of horizontal/10° of vertical/other, and major axis within 10° of maximal extension/other, for the observed data. Using likelihood χ^2^ ratio tests, we compared the main effects model, adding the two‐way interactions, and the three‐way interaction. The best‐fit model was the model with the two‐way interactions only (against main effects only, χ^2^(5) = 232.41, *p <* 0.001; against three‐way interaction, χ^2^(2) = 1.38, *p* = 0.504). Post‐hoc analysis revealed that holding the object with two hands was associated with higher proportion of views with the major axis maximally extended (40.2% vs. 31.8%), and horizontal orientation (26.0% vs. 12.3%). Finally, holding the object in the horizontal was associated with higher proportion of views with the major axis maximally extended (57.3% vs. 30.5% when vertical vs. 33% other).

In brief, the biases in infant active visual exploration of objects—use of two hands, major axis oriented perpendicular to the line of sight, and aligned with the cardinal orientations—are inter‐dependent.

## 4 Discussion

Human visual development is experience dependent. When toddlers actively engage with objects, they generate visual experiences relevant to the perception and representation of object shape. The present work shows that the generated visual experiences are highly constrained and aligned with visual sensitivities known to optimize discrimination and information extraction (Smith and Karmazyn‐Raz 2026; Wolfe and Horowitz 2004). The selectivity and specific biases in the toddler generated visual experiences also align with Marr and Nishihara's (1978) foundational ideas how object‐centric representations are formed. They proposed that some object views were better than others for forming object‐centric representations and characterized the major axis of the physical object as the “natural” axis and backbone upon which object centric representations are built. Consistent with these theoretical proposals, toddlers present themselves with a highly biased subsample of possible object views in which the extension and orientation of the major axis is tightly constrained in their egocentric view. Marr and Nishihara (1978, see also Marr 1982) also argued that views well‐structured with respect to the major axis presented non‐accidental properties of shape. Although we did not directly test this idea, the dominance of horizontal and vertical local edges in the generated views are consistent with this proposal.

We focused on the properties of toddler‐generated views because toddlerhood is a time of rapid advances object recognition and category generalization that that implicate object‐centric representations of common objects (e.g., Augustine et al. 2011; Kingo and Krøjgaard 2011; Yee et al. 2012). During this developmental period, advances in shape perception, object recognition, and category generalization have been repeatedly linked to active object manipulation. The implication, in need of a direct experimental test, is that toddler‐generated views support the rapid formation of 3D object representations. If this is correct, then the solution to how human object‐centric representations are formed may lie in the training data generated young humans.

Although there is suggestive evidence that adults may show similar biases (Ayzenberg and Behrmann 2024), it is possible that the patterns observed in toddlers are specific to young perceivers. Many machine learning studies have used toddler egocentric images of objects as training data and found advantages over other training sets for object recognition and categorization (e.g., Bambach et al. 2018; Sheybani et al. 2023; Vong et al. 2024, Ramirez et al. 2026). However, there has been limited effort directed to determining the visual properties of toddler egocentric images that are responsible for the increased efficiency in these machine learning studies. The present results provide testable hypotheses about the specific properties of training sets that may lead to the efficient formation of object‐centric representations of 3D shape for toddlers and perhaps also for machines. The prediction is that highly constrained data sets in which the major axis of the physical object often corresponds to the main axes the projected images will lead to more efficient integration of views and 3D shape representations than a complete sampling of all possible views. Such a result would challenge assumptions currently dominant in both human and machine learning that large and more diverse training sets are optimal (Bahri et al. 2024; Raviv et al. 2022; but see Zucchet et al. 2026). Often when the amount of data for training machines is larger than seems needed for human learning, theorists propose that human learning is constrained by intrinsic inductive biases that limit the conclusions that can be drawn from the noisy input (see Griffiths et al. 2010; Pouncy and Gershman 2022). The present findings suggest a different solution: that human learning may be because the training data, constrained by the learner's own behavior, is different in its structure (Ramirez et al. 2026).

### 4.1 Limitations

The results do not show that the observed properties of toddler self‐generated views actually benefit the formation of 3D shape representations. Experiments with toddlers and computational experiments that manipulate training sets and measure learning outcomes are a critical next step. The objects selected for the present study also present a limitation to the generalizability of our conclusions, albeit a potentially meaningful one. We purposely selected simple symmetrical shapes with few parts. In their simplicity, the selected objects may present the best case for views that bias the elongation of major axis. Thus, the observed biases may not hold for toddlers given more complex objects. This possibility raises the testable hypothesis that simply shaped objects could be the entry point that yields the initial discovery of an object‐centered coordinate system that is then generalized (perhaps incrementally) to more complex shapes. This idea is consistent with what is known about the long developmental path to mature visual object recognition (Ayzenberg and Behrmann 2024; Spriet et al. 2022). The results also do not identify the processes and behaviors responsible for the observed biases. The observed results implicate a role for core visual sensitivities and for acting on objects, raising several important and open questions.

### 4.2 Open Questions

A growing literature suggests that toddlers actively control the input to control attention to targets of interest and to optimize information extraction, for example, they foreground the object of interest both by leaning closer to the object and handing the object and they remove clutter, creating visual distance between the object of interest and competitors (Smith and Karmazyn‐Raz 2026; Mendez et al. 2024; Pereira et al. 2014). The present results show that toddlers generate highly varied views of objects that are organized as rotations around the longest axis of the object that is oriented horizontally or vertically. Edges and contours that are longer rather than shorter, and orientated horizontally or vertical rather than any orientation in between, attract gaze and are easier to detect and discriminate (Wolfe and Horowitz 2004), and do so from early infancy forward (see Smith and Karmazyn‐Raz 2026, for a literature review). These facts raise the hypothesis that toddlers may hold objects to enhance features that are aligned with visual sensitivities. In an early discussion of young infants’ systematic looking preferences to images with high contrast and lower spatial frequency, Banks and Ginsburg (1985) summarized the overall pattern by noting that infants systematically prefer to look at what they can see clearly. This is a general principle of human vision (Wolfe and Horowitz 2004). Freely acting toddlers can bias the visual properties of the input in multiple ways, including how they hold objects. Figure 8 illustrates this hypothesis as a control system (Brown 2006) in which toddlers actively adjust the spatial relation between sensors and object in order to optimize the extent of the object and cardinal orientations. This hypothesis requires a direct test via experiments that offer toddlers objects with stronger and weaker features that might be optimized and/or allow infants to select views by mere looking rather than through bodily actions. However, even if this hypothesis is correct, it is likely that the hands play a role in the observed biases.

**FIGURE 8:**
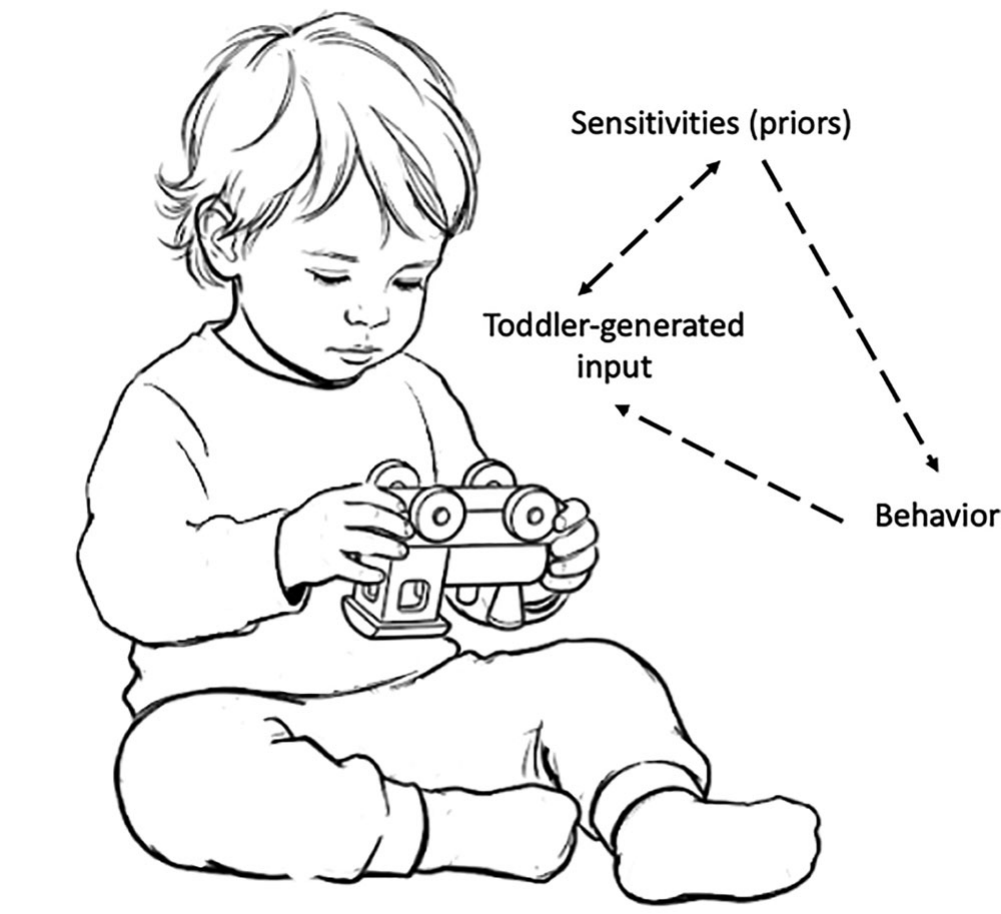
A control hypothesis. Core visual sensitivities influence behaviors and select views that align with those sensitivities, creating biased input optimized for the human visual system.

The morphology of bodies and the actions they afford are also “priors” that constrain the visual input (e.g., Bambach et al. 2016; Clark 1973; Marcinowski et al. 2019). Toddlers often engage with objects with two hands holding the object at the midline of their own body (Bambach et al. 2016). The rate of two handled‐holds centered at midline increases when postural and head stability is at risk (Corbetta and Bojczyk 2002; Hsu et al. 2016; Thompson et al. 2025) and when head stability is important to visual attention (Ruff et al. 1996; Yu and Smith 2012). The body's morphology and actions are strongly constrained by gravity, the same evolutionary force behind the visual bias for horizontal and vertical extents and edges (Clark 1973; Geisler 2008; Petroff et al. 2025). A large literature also suggest hand shapes that are designed for functionally using objects with those functions dependent on the shape (Pereira et al. 2010; Sakata et al. 1998; Smith et al. 2014). Because both human visual and motor systems evolved and develop in the same physical world of objects, the biases they create are likely aligned, interdependent, and mutually re‐enforcing (Li and Benitez 2025). Thus, visual sensitivities and bodily actions are likely jointly responsible for the present findings. These moment‐to‐moment influences need to be disentangled in experimental studies. Notably, there is evidence indicates that functional actions on objects may independently influence perceived shape (e.g., Smith 2005). In addition, haptic perception is known to directly influence visual shape representations (e.g., Jao et al. 2014). Functional actions and haptic experiences may be particularly influential in forming representations of part structure and symmetry (Smith 2005; Lockman 2000; Kyler and James 2026).

Developmental research focused on actions and the visual experiences generated by those actions is of increasing importance in the context of advancing research on the interplay of ventral and dorsal cortical pathways in shape perception, findings that counter the traditional view of specialized modules for object recognition versus vision for action (Ayzenberg and Behrmann 2022, 2024). Within this active field of current research, the present results suggest the value of increased attention to the role of low‐level visual sensitivities but also to point to the potential roles of specific object shapes in relation to grasping, handling, and action goals. Finally, the finding that toddlers—through own bodily actions—generate constrained visual experiences for object recognition has important implications for understanding atypical developmental pathways: If momentary behaviors create the momentary input and thus in aggregate the statistics of experience, then infants and children with disruptions to their sensory‐motor systems may generate disrupted inputs for visual cognition (Iverson 2021).

## Author Contributions

**Alfredo F. Pereira**: conceptualization, investigation, funding acquisition, writing – original draft, methodology, validation, writing – review and editing, software, formal analysis, supervision. **Thomas Endejan**: investigation, methodology, validation, visualization, writing – review and editing, software, formal analysis. **Linda B. Smith**: conceptualization, investigation, funding acquisition, writing – original draft, methodology, writing – review and editing, project administration, data curation, supervision, resources, formal analysis.

## Funding

The research was supported by National Institutes of Health grants R01EY032897 and R01HD104624 to Linda B. Smith and by Portuguese Foundation for Science and Technology UID/50/2025 CEECINST/00134/2021/CP2786/CT0003 (PTDC/PSI‐GER/2463/2021 to Alfredo F. Pereira).

## Ethics Statement

Recruitment of participants, consenting procedures for parents and guardians, assenting procedures for children, the protocol for data collection, data storage, and sharing the raw videos were approved by the Indiana University Review Board, Protocol # 902000021.

## Conflicts of Interest

The authors declare no conflicts of interest.

## Data Availability

The raw head camera videos are being made available on Databrary for authorized users (volume citation forthcoming). The data and all code used in the analyses, including the renditions of the handled objects and the simulated renditions for the null, are available on OSF, Pereira et al. (2026). *Visual priors in toddlers’ hands provide a foundation for 3D shape perception* [Data and software]. OSF: https://osf.io/kf8e4/overview?view_only=76606677cb944277ad28257400ccad0d. We commit to maintaining the OSF linked to this statement without modification.
